# Nicotinamide riboside combined with exercise to treat hypertension in middle-aged and older adults: a pilot randomized clinical trial

**DOI:** 10.1007/s11357-025-01815-2

**Published:** 2025-08-07

**Authors:** Yi Lin, Rola S. Zeidan, Stephanie Lapierre-Nguyen, Hannah M. Costello, Stephen D. Anton, Thomas W. Buford, Demetra D. Christou, Michelle L. Gumz, Christiaan Leeuwenburgh, Christopher R. Martens, Mary M. McDermott, Marie E. Migaud, Bhanuprasad Sandesara, Douglas R. Seals, Peihua Qiu, Yipeng Wang, Robert T. Mankowski

**Affiliations:** 1 Division of Gerontology, Geriatrics, and Palliative Care, Department of Medicine, University of Alabama at Birmingham, Birmingham, AL USA; 2 Department of Physiology and Aging, University of Florida, Gainesville, FL USA; 3 Department of Health Outcomes and Biomedical Informatics, College of Medicine, University of Florida, Gainesville, FL USA; 4 University of Colorado Anschutz Medical Campus, Aurora, CO USA; 5 Department of Applied Physiology and Kinesiology, University of Florida, Gainesville, FL USA; 6 Department of Kinesiology and Applied Physiology, University of Delaware, Delaware, DE USA; 7 Department of Internal Medicine, Northwestern University, Chicago, IL USA; 8 Department of Pharmacology, Mitchell Cancer Institute, University of South Alabama, Mobile, AL USA; 9 Department of Medicine, Division of Geriatric Medicine, University of Florida, Gainesville, FL USA; 10 Department of Integrative Physiology, University of Colorado Boulder, Boulder, CO USA; 11 Department of Biostatistics, University of Florida, Gainesville, FL USA; 12 Birmingham/Atlanta VA GRECC, Birmingham Veterans Affairs Medical Center, Birmingham, AL USA

**Keywords:** Nicotinamide riboside, Exercise, Hypertension, Older adults

## Abstract

Aerobic exercise lowers blood pressure (BP) with varying effects in hypertensive adults, potentially due to age-related nicotinamide adenine dinucleotide (NAD) metabolism dysregulation. This pilot randomized clinical trial (RCT) tested the efficacy of combining aerobic exercise with the NAD booster nicotinamide riboside (NR) to enhance BP control. In this double-blinded RCT, 54 sedentary adults (≥ 55 years) with mean daytime systolic BP (SBP) ≥ 130 mmHg were randomized to 6 weeks of 1000 mg/day of NR combined with 3 days/week of supervised 30-min walking exercise (NR + Ex), Placebo combined with the same exercise regimen (PL + Ex), or NR alone (NR). The primary outcome was daytime SBP. Other outcomes included pulse wave velocity (PWV), NAD catabolites, and nighttime BP. The primary comparison was between NR + Ex and PL + Ex. Of 54 participants (mean age 67 years, 61% female), 49 (NR + Ex: *n* = 15, PL + Ex: *n* = 16, NR: *n* = 18) completed all study visits (93% adherence to exercise and 90% to supplementation). NR + Ex (mean change = 5.19 ± 13.2 mmHg) did not reduce SBP more than PL + Ex (− 2.71 ± 10.5 mmHg). NR + Ex (− 0.31 ± 0.77 m/s) showed a trend toward a greater reduction in PWV. Levels of NAD catabolites were higher in NR groups. In a post hoc analysis, NR + Ex showed a trend toward greater nighttime BP reductions (systolic: − 9.6 ± 9.22; diastolic: − 4.51 ± 7.12 mmHg) in participants without antihypertensive medications. NR + Ex was not superior to PL + Ex in reducing BP in hypertensive middle-aged and older. However, trends toward greater nighttime BP reduction in NR + Ex in participants without antihypertensive medication warrant further investigation in a Phase IIb RCT.

**Graphical Abstract:**

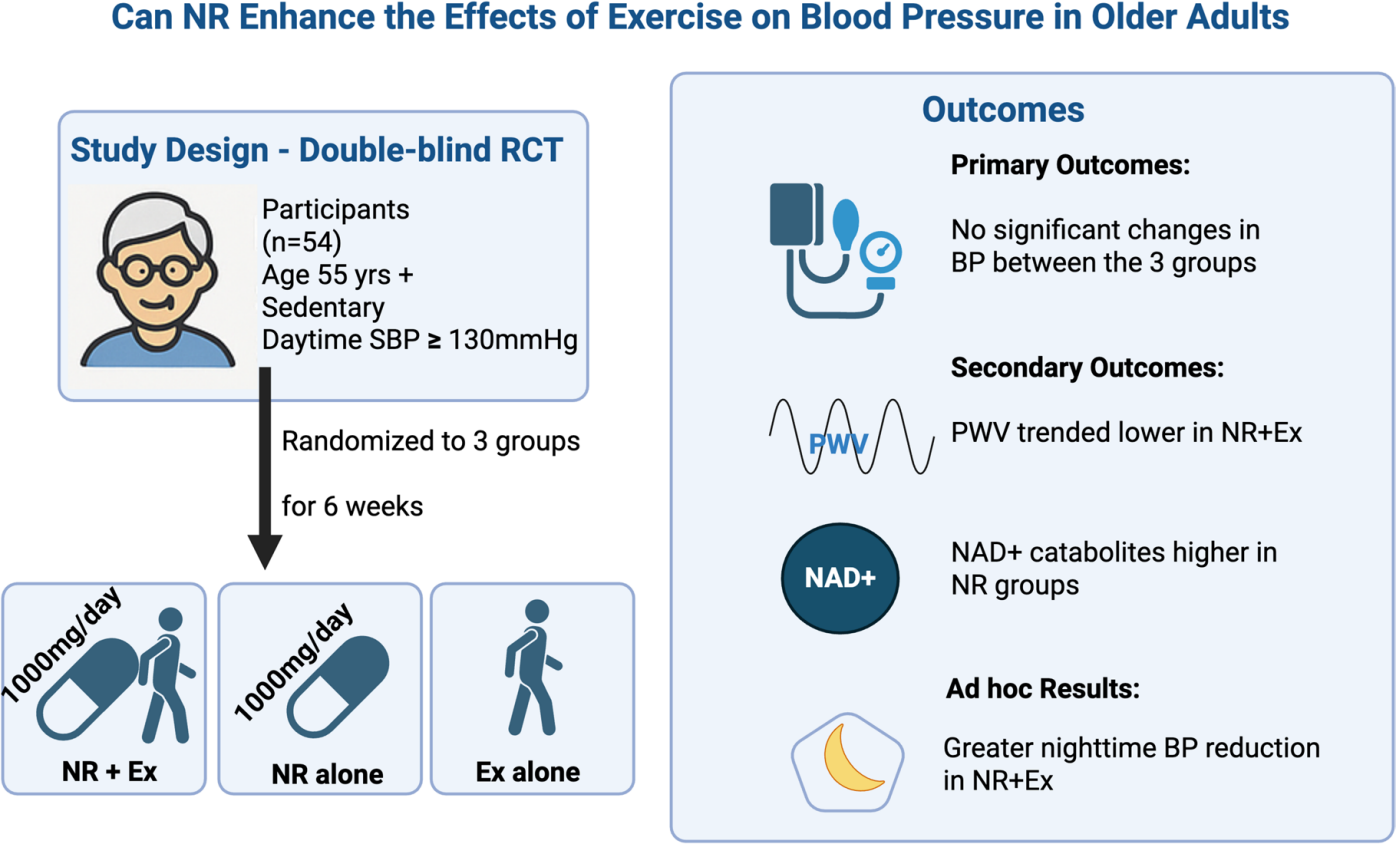

**Supplementary Information:**

The online version contains supplementary material available at 10.1007/s11357-025-01815-2.

## Introduction

Hypertension is a highly prevalent condition that becomes increasingly common with advancing age [1]. According to the American Heart Association (AHA) and the American College of Cardiology (ACC) guidelines, blood pressure (BP) > 130/80 mmHg is considered Stage I hypertension [2]. Based on these criteria, approximately 55% of middle-aged adults and over 70% of older adults (≥ 60 years old) are considered hypertensive and thus are at high risk of cardiovascular disease (CVD) events such as heart failure, myocardial infarction, and stroke [3–5]. Importantly, both daytime and nighttime systolic blood pressure (SBP) are critical factors associated with cardiovascular risk, with elevated values during either period linked to increased risk of CVD events [6, 7]. Therefore, identifying effective SBP-lowering therapies is essential for middle-aged and older adults to lower the risk of cardiovascular events and reduce mortality.

Regular aerobic exercise, such as brisk walking, has been shown to be effective in lowering SBP and improving vascular function [8, 9]. The adaptation to aerobic exercise training reduces BP partially by increasing nitric oxide (NO) bioavailability and reducing arterial stiffness, which collectively result in improved vascular function [10, 11]. However, cardiovascular function changes in older individuals in response to aerobic exercise training are variable, with some individuals not responding to exercise as expected [12]. This variability in responsiveness suggests that additional biological factors may influence individual adaptations to exercise. Age-related imbalance of the nicotinamide adenine dinucleotide (NAD +) metabolome may be a critical biological factor contributing to a variable response to aerobic exercise training in middle-aged and older adults.

NAD + is a coenzyme that functions as an electron transporter in cellular respiration and as a substrate for enzymes (e.g., the protein deacetylase sirtuin 1-SIRT1) involved in energy metabolism and cellular signaling and repair [13]. In human tissues, NAD + levels are negatively correlated with age [13], which may be caused by increased activity of NAD + -consuming enzymes in response to excessive cellular damage (e.g., poly-adenosine diphosphate ribose polymerases [PARPs] and CD38) and a reduction in nicotinamide phosphoribosyl transferase synthesis [14]. Also, reduced NAD + bioavailability blunts SIRT1 activity in older mice in response to exercise interventions, which limits the improvements in vascular function and SBP [15]. Therefore, upregulation of the NAD metabolome has the potential to enhance the effects of aerobic exercise on vascular function and optimize the management of SBP in hypertensive middle-aged and older adults.

Few biological compounds have demonstrated enhancement of cellular NAD + levels in humans [16] In the past decade, however, the compound nicotinamide riboside (NR) has been widely used as a supplement to upregulate the NAD metabolome [17, 18]. For example, 6 weeks of 1000 mg per day of oral NR supplementation was reported to safely elevate NAD + levels and reduce SBP and arterial stiffness in thirty middle-aged and older adults [19]. In the same study, post hoc subgroup analysis revealed greater SBP reductions among individuals with elevated baseline SBP, suggesting the potential for using NR supplementation to enhance vascular function in middle-aged and older adults, particularly those with elevated SBP [19]. Nonetheless, no clinical study to date has investigated the potential of NAD + boosting supplements, such as NR, for augmenting the effect of aerobic exercise on enhancing cardiovascular function and SBP control. Therefore, this pilot randomized clinical trial (RCT) aimed to provide novel information on the potential efficacy of aerobic exercise training combined with oral NR supplementation as an intervention to enhance SBP control in hypertensive middle-aged and older adults. We hypothesized that the combination of aerobic exercise and NR supplement would demonstrate potential for more effective BP control compared to either exercise with placebo or NR alone in hypertensive middle-aged and older adults.

## Methods

### Study design

We conducted a double-blinded RCT with three parallel study arms. Participants were assigned to one of the following intervention groups for 6 weeks of (1) 1000 mg/day of NR combined with 3 days/week of supervised 30-min walking exercise (NR + Ex), (2) Placebo combined with the same exercise regimen (PL + Ex), or (3) NR alone (NR). The selected NR dose and intervention duration were based on the results of prior studies [19, 20]. A dose of 1000 mg/day was reported safe and upregulated blood NAD metabolites [20], and a 6-week supplementation period reduced SBP and arterial stiffness in normotensive middle-aged and older adults [19]. This study included four in-person visits: screening, baseline, 3-week follow-up, and 6-week closeout visits. The 3-week follow-up was conducted primarily for participant safety monitoring; therefore, comparisons in this study focus on changes between baseline and the 6-week closeout. Participant safety was monitored by an interdisciplinary research team including the principal investigator, study physician, and certified research staff, with oversight from an independent Data and Safety Monitoring Board appointed by the National Institute on Aging. The study was registered at www.clinicaltrials.gov prior to participant recruitment (NCT04112043).

### Participants

This study aimed to enroll 54 sedentary adults aged ≥ 55 years old with hypertension (daytime average of SBP ≥ 130 mmHg and < 160 mmHg). Sedentary status was assessed using the Community Healthy Activities Model Program for Seniors (CHAMPS) questionnaire with < 150 min/week of moderate physical activity. Participants were excluded if they (1) regularly consumed supplements that elevate NAD + such as (niacin and vitamin B3, (2) had a Mini-Mental State Examination (MMSE) score < 24, (3) had absolute contraindications to exercise training such as uncontrolled heart failure or arrhythmias [21], or (4) had any medical conditions assessed by our study physician that would cause safety concerns.

### Recruitment, screening, randomization, and blinding

Recruitment strategies included direct mailings, newspaper advertisements, social media advertisements, printed advertisements, community luncheons, health fairs, and referrals from our Geriatric Medicine clinic. Individuals who expressed interest in the study underwent a pre-screening phone interview. Those who were deemed eligible were invited to an in-person screening visit. During this in-person screening, potential participants were asked to provide informed consent, were screened for study eligibility criteria, and completed demographic questionnaires. Screening procedures included a comprehensive review of medical history, current medications and supplement use, physical activity habits (assessed by the CHAMPS questionnaire), cognitive function assessment (assessed by the MMSE), a single daytime ambulatory SBP assessment, and a physical examination performed by the study physician. Participants who met all study entry criteria were scheduled for a baseline visit for baseline assessments and randomization into one of the three study arms.

At baseline, participants completed the following assessments: (1) 24-h ambulatory BP monitoring, (2) arterial stiffness assessment, (3) urine collection, and (4) a fasting blood draw for clinical safety laboratory panel evaluation. A research staff member not involved in direct participant interaction post-enrollment, executed the randomization procedure, prepared the study pill dispensations, and communicated the group assignment with a blinded interventionist. Randomization was performed using a random number generator and a random allocation list created by the statistician; the block randomization was stratified by age (i.e., 55–75, > 75 years) and sex. Only the study statistician and the unblinded randomizing research staff member had access to the allocation list. Research staff conducting study assessments were blinded to group assignments throughout the study.

### Assessments

The primary outcome of this study was mean daytime SBP. The secondary outcomes were diastolic BP and arterial stiffness. Additionally, urine samples were collected to analyze for NAD catabolites, and blood samples were drawn for clinical safety laboratory panels. Demographic data, anthropometrics and vitals were collected during the assessment visits.

#### Ambulatory blood pressure

All BP recordings were conducted using the validated Oscar 2™ Ambulatory Blood Pressure Monitor under the supervision of trained personnel and in accordance with the manufacturer’s recommendations [22]. SBP measurements were taken every 30 min during the day (from 07:00 to 23:00 h), and every 60 min overnight (from 23:00 to 07:00 h). Raw BP and heart rate data were stored in the device and subsequently downloaded using the AccuWin Pro™ 4 software and stored on a secured shared drive without identifier or protected health information. The software generated summary reports that included: (1) daytime and nighttime SBP and diastolic BP (DBP); (2) averages and standard deviations of each measured parameter; and (3) the total number of valid readings [22]. Ambulatory BP monitoring was performed at screening, baseline, follow-up (mid-intervention at 3 weeks), and close-out (6 weeks) visits. At each visit, trained research staff fitted participants with the BP monitor and instructed them to return the device to the research center after 24 h.

#### Arterial stiffness

Carotid-femoral pulse-wave velocity (cfPWV), the gold standard method of assessing arterial stiffness in humans [23], was used to measure aortic PWV in this study. Measurements were performed using the SphygmoCor Xcel system (AtCor Medical, New South Wales, Australia). Pressure pulse waves at the carotid and femoral arteries were assessed simultaneously. The carotid pulse was measured using the SphygmoCor tonometer and the femoral pulse was measured using a partially inflated oscillometric cuff positioned around the thigh. cfPWV was calculated as the distance between recording sites divided by the time delay between pulse wave arrivals. A trained single observer performed all baseline and 6-week closeout cfPWV measurements. The distance from the suprasternal notch to the carotid recording site and from the suprasternal notch to the femoral recording site was measured using a caliper. The distance to the carotid site was subtracted from the distance to the femoral site to determine the path length for cfPWV calculation. The average of three high-quality measures from each visit was used in the analyses [23].

#### Urine collection and analyses

Urine samples were collected at baseline and 6-week close-out visits and immediately stored in a − 80 °C freezer for further analyses. After completion of data collection, these samples were analyzed for the NAD catabolites including 1-methylnicotinamide (Me-NAM), pyridines (PY), pyridone ribosides (PYR), nicotinuric acid (NUA), N-methyl-2-pyridone-5-carboxamide (Me-PY) using mass spectrometry at the Mass Spectrometry Core Facility at the University of South Alabama [24].

#### Blood collection

At baseline, 3-week follow-up, and 6-week close-out visits, an experienced phlebotomist collected venous blood for basic safety laboratories and complete blood count. Results were processed on a rolling basis and were reviewed by a study physician and the principal investigator to monitor participant safety throughout the study.

### Interventions

#### Exercise intervention

Participants in the NR + Ex and PL + Ex study arms completed a 6-week, center-based walking exercise intervention consisting of three indoor-track walking sessions per week. Each session began with a brief 5-min warm-up followed by 30 min of walking and concluded with a 5-min cool-down period. According to the American College of Sports Medicine and American Heart Association guidelines, the intensity of walking was monitored using the Borg CR10, a Category-Ratio (CR) scale with a score of 0 (represents no physical exertion at all) to 10 (represents the extreme intensity of activity) [25]. Participants were initially instructed to walk at a moderate intensity (5–6 on the CR10 scale) and then gradually encouraged to incorporate brief periods of vigorous walking (7–8 on the CR10 scale) aiming to achieve a minimum of 10 min of vigorous walking per session. An exercise interventionist recorded the total number of laps completed on an indoor-track (54.2 m) during each 30-min session and encouraged consistent participant engagement.

#### Nicotinamide riboside (NR) supplementation

NR and matching placebo (microcrystalline cellulose) capsules for this study were provided by Niagen Bioscience (previously ChromaDex, Corp) (Los Angeles, CA). Placebo capsules were visually identical to the NR capsules. Participants were instructed to ingest 2 capsules twice a day with breakfast and dinner (total of 4 capsules a day) of NR (NIAGEN®; 250 mg, ChromaDex, Inc.) or alike-looking placebo capsules. Participants received enough capsules at the baseline visit sufficient for at least 3 weeks of supplementation. The second portion of the capsules was dispensed by trained research staff at the 3-week follow-up visit.

### Evaluation of safety and adherence

Several procedures were adopted to ensure participant safety. Trained study personnel explained the potential adverse events associated with the study activities during the informed consent process. Adverse events were recorded and monitored by research staff at each assessment visit. Exercise interventionists also documented adverse events that occurred during the supervised exercise sessions. In addition, clinical laboratory tests were performed at each assessment visit and utilized to monitor metabolic abnormalities potentially related to the interventions. The principal investigator and the study physician reviewed the reported adverse events and blood test results to ensure the participants were safe to continue participating. Study physician determined relatedness of adverse events to the study.

Adherence to the interventions was monitored throughout the study period. Participants were instructed to complete a daily diary tracking their NR supplement or placebo intake during the intervention. These diaries, along with the supplement bottles, were collected at the 3-week follow-up and the 6-week close-out visits. Research staff reviewed the returned diaries and counted the remaining capsules in each bottle to calculate compliance with NR (or placebo) intake. Additionally, exercise interventionists monitored participants’ adherence to the exercise protocol by recording attendance and Borg CR10 scale ratings (for participants’ exertion) during each session.

### Sample size and statistical analyses

Sample size consideration followed the published recommendations for study design of a phase IIa RCT [26, 27], which suggested that enrolling 15 participants per randomized arm is generally adequate to estimate parameters for a future larger-scale trial. To account for potential drop-out rate, this study aimed to recruit 18 participants for each group.

The primary comparison of interest was between participants randomized to NR + Ex and those in the PL + Ex group. The third group, which received NR without exercise, served as a secondary comparator to explore the independent effects of NR. Means and standard deviations were reported to illustrate the direction of the intervention effect on the outcomes. For primary and secondary outcomes, missing data were imputed using the mean. In the metabolite analyses, participants with missing values were excluded to preserve data integrity. Zero values were replaced with a small constant (e.g., 0.001) to prevent computational issues in rank-based analyses or other calculations for metabolite analyses. To compare baseline characteristics and safety-related blood chemistry between groups, the normality of continuous variables was assessed using the Shapiro–Wilk test; normally distributed data were analyzed with one-way ANOVA, while non-normally distributed variables were assessed using the Kruskal–Wallis test. Principal component analysis was used to identify major patterns of variance across variables, and the resulting components were used to guide post hoc subgroup analyses. All statistical analyses were conducted using R (version 4.4.3) and IBM SPSS Statistics (version 30).

#### Study approval

This study was approved by the University of Florida’s Institutional Review Board (IRB201900746). All participants provided written informed consent prior to enrollment, in accordance with the Declaration of Helsinki.

## Results

### Participants

**Fig. 1:**
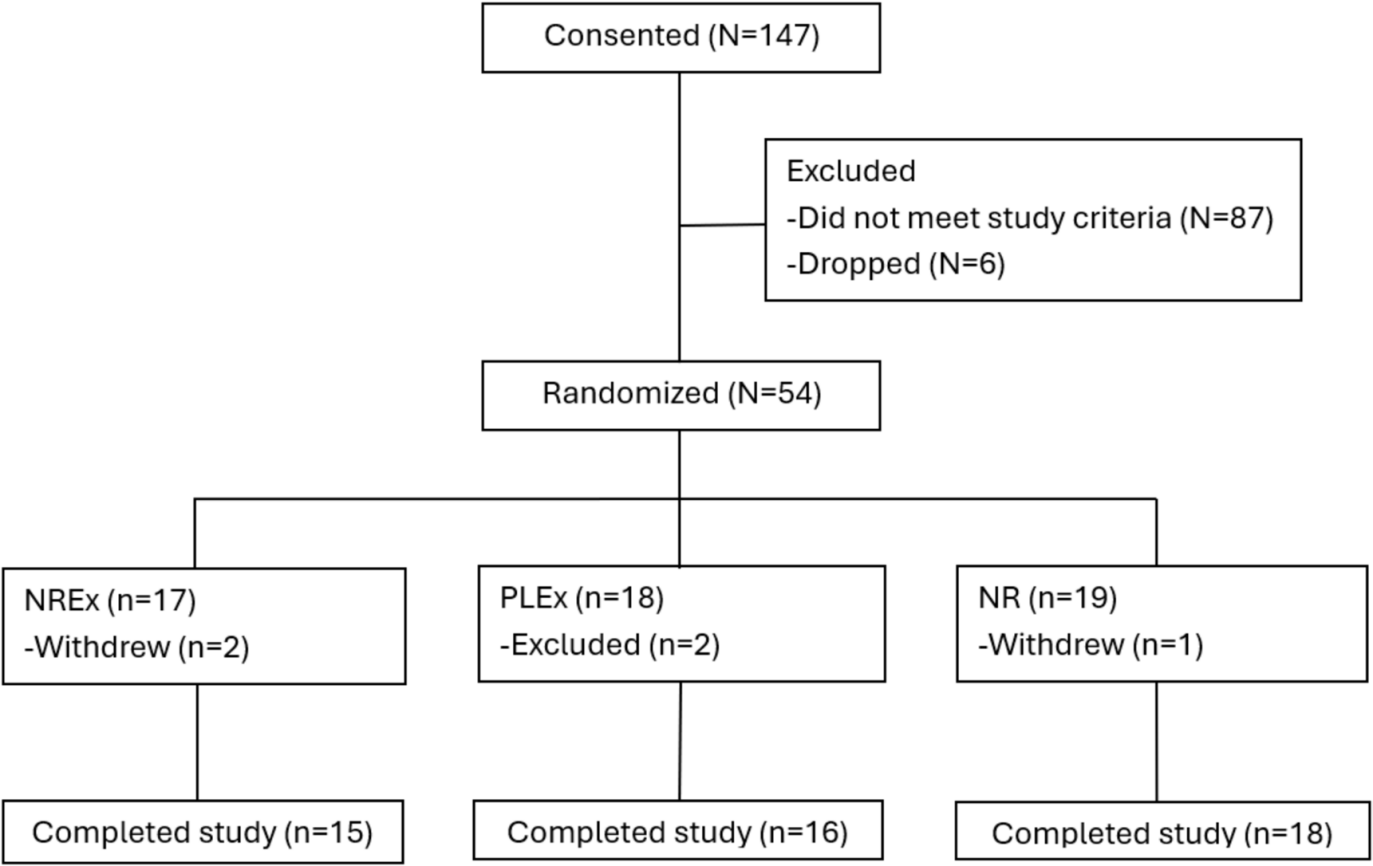
CONSORT diagram

Fifty-four sedentary, hypertensive older adults were randomized to one of the three 6-week intervention groups (NR + Ex: *n* = 17, PL + Ex: *n* = 18, NR: *n* = 19). Two participants were excluded after randomization due to personal conditions that prevented them from participating in exercise training, and three withdrew during the study period due to discomfort from wearing a 24-h ambulatory BP monitor or personal reasons. Of the remaining participants, 50 completed the assigned interventions, and 49 (NR + Ex: *n* = 15, PL + Ex: *n* = 16, NR: *n* = 18) completed all study assessments and follow-up visits (Fig. 1). Eighteen participants did not use antihypertensive medications, while others used antihypertensive medications spanning six major classes, including Angiotensin-converting enzyme inhibitors, angiotensin II receptor blockers, beta blockers, calcium channel blockers, diuretics, and alpha blockers. Baseline characteristics of participants across the three intervention groups are summarized in Table 1. No significant differences were found among the groups for any baseline variables.

**Table 1:** Baseline characteristics (*n* = 54)

	NR + Ex (*n* = 17)	PL + Ex (*n* = 18)	NR (*n* = 19)	*p*-value
Age (years)	67.5(7.0)	68.3(7.7)	66.3(7.2)	0.513
Sex (% female)	65	56	63	NA
Race (% Black)	29	22	32	NA
Education (% college education and above)	94	100	95	NA
Weight (kg)	91.6(19.3)	84.7(17.8)	88.4(23.7)	0.590
BMI (kg/m^2^)	33.0(7.2)	30.8(5.7)	30.7(7.8)	0.631
CHAMPS (minutes)	32.1(44.3)	22.8(30.9)	44.8(55.2)	0.662
MMSE	27.8(2.2)	28.4(1.5)	28.1(1.7)	0.787
Daytime SBP (mmHg)	139.6(12.4)	141.0(11.6)	138.9(9.9)	0.854
Daytime DBP (mmHg)	76.5(9.8)	76.0(7.0)	77.8(8.7)	0.816
PWV (m/s)	8.8(1.2)	8.7(1.4)	8.7(1.4)	0.937
# of Medications	7.2(4.2)	5.6(3.1)	6.7(4.0)	0.574
# of Antihypertensive Medications	1.1(1.0)	1.4(1.2)	1.2(1.2)	0.720

Note: Mean (Standard Deviation); *BMI* Body Mass Index, *CHAMPS* Community Healthy Activities Model Program for Seniors questionnaire, *MMSE* Mini-Mental State Examination, *SBP* systolic blood pressure, *DBP* diastolic blood pressure, *PWV* pulse-wave velocity, *NR* + *Ex* NR combined with exercise, *PL* + *Ex* placebo combined with exercise; *p*-value was derived from comparisons of the mean value between groups, statistical significance (*p* < 0.05) marked with asterisk (*)

### Retention

The overall participant retention rate was 91%, defined as the proportion of individuals who were randomized to an intervention, attended study visits, and completed all outcome measures.

### Adherence

One participant withdrew from the study after randomization due to discomfort from wearing the 24-h ambulatory BP monitor, citing excessive cuff pressure and noise as the primary reasons. Among the 50 participants who completed the intervention, adherence to NR or placebo supplementation was 90%. Exercise adherence among the 32 participants assigned to an exercise group (NR + Ex and PL + Ex) was 93%.

### Outcome measures

#### Ambulatory blood pressure

Participants in the PL + Ex group exhibited a trend toward a greater reduction after intervention in daytime SBP (− 2.71 ± 10.5 mmHg, Cohen’s *d* =  − 0.259) compared to those in the NR group (− 0.48 ± 8.49 mmHg, *d* =  − 0.057), whereas the NR + Ex group showed a trend toward an increase in daytime SBP (5.19 ± 13.2 mmHg, *d* = 0.391). In addition, participants in the PL + Ex group exhibited a trend toward a greater reduction after intervention in daytime DBP (− 1.9 ± 4.98 mmHg, *d* =  − 0.381) compared to those in the NR group (− 1.73 ± 6.67 mmHg, *d* =  − 0.259), whereas the NR + Ex group showed a trend toward an increase in daytime DBP (0.469 ± 8.01 mmHg, *d* = 0.059). Mean values and standard deviations at baseline and closeout visits are presented in **Supplement 1**.

#### Arterial stiffness

Participants in NR + Ex exhibited a trend toward a greater reduction after intervention in PWV (− 0.31 ± 0.77 m/s, *d* =  − 0.403) compared to those in the PL + Ex group (− 0.11 ± 0.78 m/s, *d* =  − 0.143), whereas the NR group showed a trend toward an increase in PWV (0.03 ± 0.9 m/s, *d* = 0.029). Mean values and standard deviations at baseline and closeout visits are presented in **Supplement 1**.

#### NAD catabolites in urine

A total of 44 viable samples were included in analyses of urine catabolite (NR + Ex: *n* = 12, PL + Ex: *n* = 15, NR: *n* = 17). PL + Ex group showed decreased Me-NAM levels (− 0.02 ± 0.04 uM/uM, *d* =  − 0.358) after intervention, while both NR + Ex (0.43 ± 0.33 uM/uM, *d* = 1.31) and NR (0.65 ± 0.87 uM/uM, *d* = 0.747) groups showed an increase. Similarly, for PY, PL + Ex showed decreased PY levels (− 0.01 ± 0.05 uM/uM, *d* =  − 0.284) after intervention, while both NR + Ex (0.03 ± 0.04 uM/uM, *d* = 0.685) and NR (0.03 ± 0.03 uM/uM, *d* = 1.15) groups showed an increase. Only the NR group showed a decrease in PYR (− 0.09 ± 0.29 uM/uM, *d* =  − 0.324) and NUA (− 0.3 ± 2.92 uM/uM, d =  − 0.103) levels after intervention. All three groups exhibited an increase in Me-PY level after intervention, with the NR + Ex group (9.18 ± 8.07 uM/uM, *d* = 1.14) increasing the most. Mean change values (closeout minus baseline), standard deviations, and effect size for all catabolites are presented in Table 2.

**Table 2:** NAD + urine catabolites

	NR + Ex (*n* = 12)		PL + Ex (*n* = 15)		NR (*n* = 17)	
Catabolites	Mean (SD)	Cohen’s d	Mean (SD)	Cohen’s d	Mean (SD)	Cohen’s d
*1-methylnicotinamide (*Me-NAM)	0.43(0.33)	1.31	− 0.02(0.04)	− 0.358	0.65(0.87)	0.747
*pyridines (*PY)	0.03(0.04)	0.685	− 0.01(0.05)	− 0.284	0.03(0.03)	1.15
*pyridone ribosides (*PYR)	0.004(0.5)	0.008	0.22(0.92)	0.236	− 0.09(0.29)	− 0.324
*nicotinuric acid (*NUA)	0.1(4.33)	0.023	0.07(0.81)	0.085	− 0.3(2.92)	− 0.103
*N-methyl-2-pyridone-5-carboxamide (*Me-PY)	9.18(8.07)	1.14	0.23(0.62)	0.377	6.93(5.08)	1.36

Note: unit = uM/uM; *NR* + *Ex* NR combined with exercise, *PL* + *Ex* placebo combined with exercise; *SD* standard deviation; mean change calculated with 6-week value minus baseline

### Post hoc analysis of 24-h BP and urine catabolite data

Principal component analysis was conducted using baseline variables including age, sex, antihypertensive medication use, CHAMPS physical activity score, MMSE score, and daytime SBP. The first two principal components (PC1 and PC2) explained 43% of the total variance, with PC1 accounting for 22% and PC2 for 21%. Antihypertensive medication use demonstrated strong contributions to both PC1 (loading = 0.51) and PC2 (loading = 0.32), suggesting it as a primary confounding factor. Other variables such as daytime SBP, MMSE, and CHAMPS also demonstrated moderate contributions to the first two components. These results suggest that antihypertensive medication use may be an important differentiating factor and provide a basis for a post hoc subgroup analysis when evaluating BP and metabolite outcomes among participants who were not using hypertensive medications. Having 24-h ambulatory BP recordings, we evaluated both the daytime and nighttime BP in this subanalysis.

#### Participant characteristics in a non-antihypertensive medication subset

A total of 18 participants (NR + Ex: *n* = 6, PL + Ex: *n* = 4, NR: *n* = 8) were not taking antihypertensive medication before and during the intervention period. No significant differences in baseline characteristics were observed among the intervention groups within the non-users of antihypertensive medication subgroup. Baseline characteristics for participants without using antihypertensive medication are presented in Supplement 2.

#### Daytime and nighttime ambulatory blood pressure

Among participants not using antihypertensive medications (*n* = 18), both the NR + Ex (mean change =  − 3.16 ± 11.7 mmHg, Cohen’s d =  − 0.27) and PL + Ex (− 11.6 ± 5.08 mmHg, *d* =  − 2.29) groups showed trends toward reductions in daytime SBP. For nighttime SBP, participants not using antihypertensive medication in NR + Ex group showed greater trends toward decreases (− 9.6 ± 9.22 mmHg, *d* =  − 1.04) compared to PL + Ex (− 1.17 ± 28.2 mmHg, *d* =  − 0.041) group.

Daytime DBP decreased in both NR + Ex (− 3.02 ± 5.7 mmHg, *d* =  − 0.531) and PL + Ex (− 6.03 ± 1.57 mmHg, *d* =  − 3.83) among participants not using antihypertensive medication. For nighttime DBP, participants not using antihypertensive medications showed reductions across all groups, with NR + Ex showing the greatest decrease (− 4.51 ± 7.12 mmHg, *d* =  − 0.633) compared to PL + Ex (− 1.06 ± 9.7 mmHg, *d* =  − 0.109) and NR group (− 0.92 ± 5.81 mmHg, *d* =  − 0.159). Mean values and standard deviations at baseline and closeout visits are presented in Supplement 3.

#### NAD urine catabolites

Among participants not taking antihypertensive medications, both the NR + Ex (mean change = 0.28 ± 0.31 uM/uM, *d* = 0.892) and NR (0.9 ± 0.97 uM/uM, *d* = 0.922) groups exhibited increases in Me-NAM levels. In contrast, the PL + Ex group showed a decrease (− 0.05 ± 0.09 uM/uM, *d* =  − 0.557). Similarly, for PY, increases were observed in both NR + Ex (0.01 ± 0.02 uM/uM, *d* = 0.617) and NR (0.04 ± 0.03 uM/uM, *d* = 1.39) among participants not using antihypertensive medications, while the PL + Ex group showed a decrease (− 0.05 ± 0.09 uM/uM, *d* =  − 0.578).

For PYR, only the NR alone group demonstrated a decrease among participants not taking antihypertensive medications (− 0.05 ± 0.11 uM/uM, *d* =  − 0.484). For NUA, reductions were noted in NR + Ex (− 2.31 ± 3.77 uM/uM, *d* =  − 0.612) and NR (− 0.1 ± 0.5 uM/uM, *d* =  − 0.198) among participants not taking antihypertensive medications. Finally, Me-PY levels increased across all groups among participants not taking antihypertensive medications, with the NR group exhibiting the largest increase (6.2 ± 2.95 uM/uM, *d* = 2.1).

### Adverse events

Among the middle-aged and older adult participants, the combined NR and aerobic exercise intervention did not indicate any abnormalities in blood laboratory results. While individual data are not shown, group means are presented in Supplement 4.

One participant in the NR only group reported a serious adverse event that was determined as unrelated to study participation by the study physician. Additionally, mild adverse events either related or unrelated to the study were reported during the study period by nine participants in the NR + Ex group, twelve participants in the PL + Ex group, and seven participants in the NR group. Study-related symptoms included dizziness, shortness of breath, fatigue, and low BP after exercise. No participants withdrew from the study due to adverse effects related to the intervention.

## Discussion

To our knowledge, this was the first pilot study to test the efficacy of a combined effect of NR supplementation with aerobic exercise training on ambulatory 24-h BP and urine NAD catabolites in middle-aged and older adults. This pilot aimed to obtain information on the efficacy of the intervention and variance for a sample size calculation for a Phase IIb RCT. We found that NR + Ex was not more effective than exercise alone in controlling BP, but a post hoc analysis indicated that NR + Ex may have potential benefits on nighttime BP control among hypertensive participants not using BP medications.

Our study demonstrated 91% retention rate among randomized participants. This level of retention is similar to that reported in other trials involving NR supplementation [18, 19, 28], particularly given that our study uniquely incorporated a supervised aerobic exercise intervention three times per week in combination with NR. In addition to successful data collection across all study visits, participants showed high adherence to both the supplement and exercise protocols, and there were no withdrawals related to the intervention. These outcomes indicate strong participant engagement. The follow-up strategies used in this study, including regular phone calls, a three-week safety follow-up visit, and consistent encouragement from the exercise interventionist, likely contributed to the high retention and adherence rates. Study product, NR, was well tolerated, with no abnormal values observed in safety-related blood chemistry measures. Reported adverse events were mild and the incidence was comparable across intervention groups. Our study results along with the overall safety profile align with previous study results and support NR use in future trials assessing its impact on vascular and metabolic health in middle-aged and older adults [19]. These findings from our unique design support implementing similar combined interventions in future larger-scale trials.

As expected, given the pilot nature of this study, ambulatory BP (systolic and diastolic) and arterial stiffness (measured by PWV) did not show statistically significant changes among groups, indicating no significant effect for 6-week NR supplementation, with or without exercise on BP or overall vascular health. Participants in the PL + Ex group exhibited a trend for a reduction in SBP compared to the NR + Ex and NR groups, suggesting that exercise alone may have a more pronounced effect on BP. It is also worth noting that the NR + Ex group started with a lower average SBP at baseline, which may have limited the observable intervention effect within this group [19]. Similarly, no significant differences in PWV were observed among the 3 intervention groups, indicating that the interventions had minimal impact on arterial stiffness over the 6-week study period. Besides a small sample size, this could potentially be attributed to the short period (6 weeks) of the intervention, especially since other studies have suggested longer intervention periods [29].

Our results are not unexpected, as prior studies—though inconsistent in their findings—generally did not demonstrate a significant effect on BP. A study by Martens et al. reported a non-statistically significant decrease in both systolic and diastolic BP after a 6-week 500 mg NR intervention, especially for SBP that was reduced on average by 9 mmHg in those with hypertension but not in those with normal baseline SBP. Another group reported positive effects of administering NR (250 mg and not 500 mg) combined with a polyphenol (pterostilbene) for 8 weeks on diastolic BP [30]; however, these results can be attributed to the pterostilbene use [31]. Notably, several other NR supplementation studies using different NR doses (ranging from 100 to 2000 mg) and intervention times (8 days to 12 weeks), found no effect of NR on BP [17, 32–34]. Taken together, these findings reinforce the emerging consensus that NR supplementation alone may not significantly influence BP or arterial stiffness, particularly over short durations, and highlight the need for longer-term studies or combination interventions to better assess potential vascular benefits.

Additionally, distinct catabolite responses to NR supplementation were observed, indicating its bioactivity. Specifically, increases in Me-NAM, PY, and Me-PY levels suggest that NR was effectively metabolized, confirming its potential role in influencing metabolic pathways. NAM is released from NAD + upon consumption of NAD + by enzymes activated upon oxidative stress (e.g., PARPs). NAM is either recycled to NAD or methylated to methyl-nicotinamide (Me-NAM), or oxidized to a pyridone (PY) or N-oxide pyridine. Once methylated, Me-NAM is readily oxidized to the methyl-pyridones (Me-PY). These urinary catabolites report on the extent of the daily systemic NAD + turnover and overall consumption [35]. However, the implications of these metabolite changes for clinical outcomes require further investigation.

Significant group differences in metabolites, including Me-NAM, PY, and Me-PY, suggest that NR exhibits an effect on metabolic processes mainly related to NAD⁺ biosynthesis. NR supplementation in both the NR + Ex and NR groups indicated an increase in Me-NAM levels. Remarkably, studies have indicated that NR supplementation is indeed associated with higher Me-NAM levels which is a metabolite of NAM (a metabolite produced by NAD + breakdown) [36, 37]. Higher PY and Me-PY, which can also be produced as a result of NAD + metabolism) indicates accelerated metabolism and clearance of NAD +, suggesting an increased processing of NAD +, knowing that NR is an NAD + precursor. Notably, several previous studies suggested that NR supplementation can increase NAD + [19, 38, 39]. Higher levels of these metabolites in the NR + Ex and NR groups compared to the PL + Ex group provide biochemical evidence supporting NR absorption and metabolism. These findings highlight the potential of NR supplementation to influence metabolic pathways, which may have implications for cellular energy metabolism and aging-related processes. We currently cannot make a mechanistic link between NAD catabolites and BP regulation, and thus future studies warrant measuring NAD metabolism in both urine and blood, to make a closer mechanistic connection with BP control.

On the other hand, despite NR’s role in boosting NAD metabolism and exercise’s known effects on mitochondrial function, this study found no additive or independent effect of NR or exercise on NAD catabolism (NUA) or mitochondrial-related metabolites. Also, differences in metabolite levels between antihypertensive medicated and non-medicated participants suggest that antihypertensive medications may influence NR metabolism or downstream NAD metabolic pathways.

Our post hoc analysis identified antihypertensive medication use as a potential confounding factor in BP and catabolite responses to NR + Ex. A subgroup analysis revealed differential effects of NR and exercise on BP depending on medication status. Among participants not taking antihypertensive medications, the PL + Ex group exhibited the greatest reductions in daytime SBP, whereas the NR + Ex group exhibited the greatest reductions in nighttime SBP. These findings suggest that antihypertensive medication status may influence the effectiveness of NR and exercise interventions on cardiovascular outcomes. While no previous studies have investigated the combined effects of antihypertensive medication and NR supplementation on BP control in hypertensive older adults, research indicates a potential negative effect of certain anti-hypertensive medications on exercise performance [40]. Therefore, the next potential step in this research line could be testing the effects of NR + Ex among middle-aged and older adults with elevated BP who are not on BP medications, in order to test whether BP medications are a potential confounding factor in this interventional approach. According to the AHA/ACC guidelines [41], individuals who have elevated BP and are not treated with antihypertensive medication are recommended for lifestyle modification such as exercise and diet for about 3 months to control BP, before starting antihypertensive medication therapy.

Additionally, the focus of further investigation may be the effect of NR + Ex on nighttime BP. Nighttime BP, including reduced nighttime dipping, is a stronger predictor of CVD and mortality than daytime BP, as it better reflects basal BP without fluctuations from physical activity and emotional stress. Elevated nighttime BP (> 110/65 mmHg) and reduced dipping have been associated with greater occurrence of cardiovascular events such as atherosclerotic CVD, stroke, and heart failure [42, 43].

As for the NAD catabolite analyses, NR and NR + Ex supplementation led to notable increases in Me-NAM and PY levels among participants not taking antihypertensive medications. This suggests enhanced NAD metabolism in the absence of medication interference. While similar trends were observed in medicated participants, the effects appeared attenuated, indicating that antihypertensive drugs may modulate the metabolic response to NR supplementation.

## Study strengths and limitations

A key strength of this study is the high retention and adherence rates, and low adverse event rates. Additionally, the inclusion of both clinical assessments of BP and urine NAD catabolites provides an evaluation of NR effects to give a strong basis for comprehensive evaluation of NAD metabolites in whole blood and catabolites in urine in a larger-scale RCT. The use of validated ambulatory BP monitoring provided a reliable recruitment of hypertensive older adults as well as sensitive continuous BP assessments accounting for 24-h BP level fluctuations. Given the nature of a pilot study, we aimed to test a short duration (6 weeks) intervention on a relatively small sample size in order to obtain preliminary efficacy data to be tested in a future fully powered RCT with a longer intervention duration (12 weeks).

## Conclusions

To our knowledge, this was the first pilot study to test the preliminary efficacy of a combined effect of NR + Ex on BP and NAD catabolites in middle-aged and older adults with hypertension. We found that NR + Ex did not demonstrate trends toward greater efficacy than aerobic exercise alone in the main cohort. However, the hypothesis-generating results of the the post hoc analysis indicated that NR + Ex may have potential benefits on nighttime BP control among hypertensive participants not using BP medications, which warrant testing NR + Ex in a fully powered RCT in participants without antihypertensive medications. Since previous research has shown that nighttime BP is a more reliable measure of resting BP and a better predictor of cardiovascular events, testing efficacy of NR + Ex on nighttime BP control warrants further investigation.

## Supplementary Information

Below is the link to the electronic supplementary material.

Supplementary file1 (DOCX 33 KB)

## Data Availability

The data are not publicly available due to ethical restrictions related to participant confidentiality. Deidentified data are available from the corresponding author upon reasonable request, with submission of an appropriate research plan.
