# Supplementary material for: Nicotinamide riboside combined with exercise to treat hypertension in middle-aged and older adults: a pilot randomized clinical trial
Source: GeroScience. 2025 Aug 7;47(6):6895–908. doi: 10.1007/s11357-025-01815-2 (PMC12638573; doi:10.1007/s11357-025-01815-2)
Supplement: Supplementary file 1 — Supplementary file1 (DOCX 33 KB) [file 11357_2025_1815_MOESM1_ESM.docx]

**NEET Supplementary Material**

**Supplement 1.** **Mean values and standard deviations at baseline and closeout visits**

|  | NR+Ex (n=15) | | PL+Ex (n=16) | | NR (n=18) | |
| --- | --- | --- | --- | --- | --- | --- |
| Visit Time | Baseline | 6-week | Baseline | 6-week | Baseline | 6-week |
| Daytime SBP | 141(12.6) | 146(14.3) | 141(12.3) | 138(14.9) | 138(9.9) | 138(7.0) |
| Daytime DBP | 78.1(8.9) | 78.6(11.2) | 75.7(7.3) | 73.8(9.8) | 78.4(8.4) | 76.6(7.0) |
| PWV | 8.8(1.3) | 8.5(1.0) | 8.6(1.2) | 8.5(1.1) | 8.7(1.4) | 8.8(1.5) |

*Note: Mean (Standard Deviation); SBP: Systolic Blood Pressure; DBP: Diastolic Blood Pressure; PWV: Pulse-Wave Velocity; NR+Ex: NR combined with exercise; PL+Ex: placebo combined with exercise*

**Supplement 2. Baseline characteristics – participants without BP medications (n = 18)**

|  | NR+Ex  (n=6) | PL+Ex  (n=4) | NR  (n=8) | *p*-value |
| --- | --- | --- | --- | --- |
| Age (years) | 65.3(8.2) | 68.8(11.4) | 69.8(5.3) | 0.580 |
| Sex (female%) | 33 | 75 | 63 | NA |
| Race (% Black) | 17 | 0 | 0 | NA |
| Education (% college education and above) | 100 | 100 | 88 | NA |
| Weight (kg) | 90.1(10.0) | 76.3(18.5) | 80.5(21.1) | 0.443 |
| BMI (kg/m^2^) | 30.3(4.7) | 29.4(4.8) | 28.6(6.9) | 0.867 |
| CHAMPS (minutes) | 40(59.0) | 22.5(28.7) | 67.1(74.5) | 0.769 |
| # of Medications | 6.5(5.6) | 3.0(2.6) | 5.8(5.8) | 0.622 |
| # of Antihypertensive Medications | 0(0) | 0(0) | 0(0) | NA |
| MMSE | 28.2(2.1) | 29.0(0.8) | 28(1.8) | 0.743 |
| Daytime SBP (mmHg) | 142.2(8.0) | 143.3(6.9) | 135.0(6.2) | 0.101 |
| Daytime DBP (mmHg) | 77.5(7.9) | 77.1(6.3) | 75.8(7.2) | 0.895 |
| PWV (m/s) | 8.4(0.4) | 7.9(1.8) | 8.2(1.7) | 0.857 |
| Nighttime SBP (mmHg) | 132.3(17.7) | 132.3(19.1) | 118.2(10.5) | 0.177 |
| Nighttime DBP (mmHg) | 70.3(9.9) | 65.7(5.7) | 65.0(8.6) | 0.509 |

*Note: Mean (Standard Deviation); BMI: Body Mass Index; CHAMPS: Community Healthy Activities Model Program for Seniors questionnaire; MMSE: Mini-Mental State Examination; SBP: Systolic Blood Pressure; DBP: Diastolic Blood Pressure; PWV: Pulse-Wave Velocity NR+Ex: NR combined with exercise; PL+Ex: placebo combined with exercise;* *p-value was derived from comparisons of the mean value between groups, statistical significance (p<0.05) marked with**

**Supplement 3. Baseline and closeout values for participants not using antihypertensive medications**

|  | NR+Ex (n=6) | | PL+Ex (n=4) | | NR (n=8) | |
| --- | --- | --- | --- | --- | --- | --- |
| Visit Time | Baseline | 6-week | Baseline | 6-week | Baseline | 6-week |
| Daytime SBP | 142(8) | 139(7.2) | 143(6.9) | 132(4.2) | 135(6.2) | 138(6.7) |
| Nighttime SBP | 132(17.7) | 123(11.5) | 132(19.1) | 131(18.3) | 118(10.5) | 119(14.3) |
| Daytime DBP | 77.5(7.9) | 74.5(8.5) | 77.1(6.3) | 71.1(7.3) | 75.8(7.2) | 77.6(6.0) |
| Nighttime DBP | 70.3(9.9) | 65.8(10.7) | 65.7(5.7) | 64.6(6.1) | 65(8.6) | 64.1(11) |

*Note: Mean (Standard Deviation); SBP: Systolic Blood Pressure; DBP: Diastolic Blood Pressure; NR+Ex: NR combined with exercise; PL+Ex: placebo combined with exercise*

**Supplement 4. Safety blood chemistries at baseline, 3 and 6 weeks**

|  | NR+Ex (n=15) | | | PL+Ex (n=16) | | | NR (n=18) | | |  |
| --- | --- | --- | --- | --- | --- | --- | --- | --- | --- | --- |
| Test | Baseline | 3-week | 6-week | Baseline | 3-week | 6-week | Baseline | 3-week | 6-week | p-value |
| Sodium | 139(1.9) | 140(3.1) | 139(2.2) | 140(1.8) | 141(2.3) | 140(2.1) | 140(3.0) | 140(3.2) | 140(2.7) | 0.908 |
| Potassium | 4.1(0.3) | 4.1(0.2) | 4.2(0.3) | 4.2(0.3) | 4.2(0.3) | 4.2(0.3) | 4.3(0.4) | 4.2(0.3) | 4.2(0.3) | 0.541 |
| Chloride | 103(2.3) | 103(2.6) | 103(2.3) | 103(2.2) | 104(2.7) | 104(2.6) | 104(3.3) | 104(3.1) | 104(2.8) | 0.071 |
| CO2 | 27.7(2.1) | 27(1.9) | 27.4(2.0) | 28.4(2.5) | 28.8(2.5) | 27.9(2.5) | 27.6(2.7) | 27.4(2.7) | 27.5(2.8) | 0.998 |
| Urea Nitrogen | 17(4.2) | 17(5.1) | 15.2(5.4) | 15.5(6.0) | 16.9(7.0) | 15.4(6.3) | 14.8(4.3) | 14.3(3.9) | 15.4(5.0) | 0.461 |
| Creatinine | 0.9(0.1) | 0.9(0.1) | 0.9(0.1) | 1.1(0.4) | 1.1(0.4) | 1.0(0.3) | 0.9(0.2) | 0.9(0.2) | 0.9(0.2) | 0.574 |
| BUN/Creatinine Ratio | 19.3(5.1) | 18.9(5.5) | 17.6(7.2) | 15(2.9) | 16.6(5.0) | 15.2(4.1) | 17(6.2) | 16.6(5.6) | 17.4(5.7) | 0.337 |
| Glucose | 114(43.5) | 110(35.0) | 118(39.5) | 106(30.8) | 105(23.1) | 107(30.7) | 121(68.0) | 124(76.2) | 119(62.7) | 0.552 |
| Calcium | 9.6(0.5) | 9.6(0.5) | 9.6(0.4) | 9.4(0.5) | 9.3(0.4) | 9.3(0.4) | 9.4(0.4) | 9.4(0.3) | 9.4(0.4) | 0.490 |
| Total Protein | 7.3(0.5) | 7(0.5) | 7.1(0.5) | 7(0.7) | 6.9(0.6) | 6.8(0.6) | 7.2(0.3) | 7.2(0.3) | 7.3(0.4) | 0.125 |
| Albumin | 4.4(0.2) | 4.2(0.7) | 4.3(0.2) | 4.2(0.3) | 4.2(0.3) | 4.2(0.3) | 4.3(0.2) | 4.3(0.2) | 4.3(0.2) | 0.624 |
| Calc Total Globulin | 2.9(0.4) | 2.7(0.4) | 2.8(0.4) | 2.8(0.6) | 2.7(0.5) | 2.6(0.5) | 2.9(0.3) | 2.9(0.3) | 3(0.4) | 0.088 |
| Albumin/Globulin Ratio | 1.6(0.2) | 1.7(0.3) | 1.6(0.3) | 1.6(0.3) | 1.6(0.3) | 1.7(0.4) | 1.5(0.2) | 1.5(0.2) | 1.5(0.2) | 0.163 |
| Total Bilirubin | 0.6(0.2) | 0.7(0.3) | 0.7(0.3) | 0.6(0.2) | 0.6(0.2) | 0.6(0.1) | 0.6(0.2) | 0.6(0.3) | 0.6(0.2) | 0.731 |
| Alkaline Phosphatase | 73.1(13.8) | 71.3(13.9) | 71(14.7) | 69.8(23.1) | 69.6(24.6) | 70.2(24.2) | 68.3(21.3) | 68.5(20.1) | 71.8(29.1) | 0.279 |
| AST | 21.4(9.0) | 19.1(5.9) | 18.8(4.3) | 19.8(6.8) | 20.1(6.1) | 18.8(4.3) | 23.5(14.5) | 20.6(11.1) | 20.4(4.4) | 0.795 |
| ALT | 19.6(10.4) | 16.8(6.0) | 16.7(6.4) | 14.9(5.0) | 19.2(10.5) | 16.9(8.3) | 23(17.4) | 21.1(15.5) | 18.9(8.7) | 0.054 |
| Anion Gap | 8.7(1.7) | 9.3(1.8) | 8.5(1.9) | 8.7(2.0) | 8.1(1.8) | 8.2(1.8) | 8.7(2.1) | 8.2(2.1) | 9.3(2.2) | 0.384 |
| WBC | 5.4(1.5) | 5.3(1.7) | 5.2(1.4) | 6.1(1.2) | 5.9(1.3) | 5.9(1.2) | 5.9(1.7) | 5.3(1.2) | 5.7(2.0) | 0.913 |
| RBC | 4.6(0.4) | 4.6(0.4) | 4.5(0.4) | 4.7(0.6) | 4.7(0.7) | 4.7(0.7) | 4.6(0.5) | 4.6(0.4) | 4.7(0.5) | 0.134 |
| Hemoglobin | 13.7(1.0) | 13.5(0.9) | 13.4(1.3) | 13.3(1.1) | 13(1.2) | 13.1(1.1) | 13.6(1.2) | 13.5(0.9) | 13.7(1.2) | 0.203 |
| Hematocrit | 40.7(2.8) | 40(2.5) | 39.8(3.4) | 39.7(3.4) | 39(3.6) | 39.3(3.4) | 40.4(3.3) | 40(2.5) | 40.6(3.3) | 0.269 |
| MCV | 88.1(5.4) | 88.2(5.9) | 88.3(6.0) | 84.8(7.5) | 84.8(8.0) | 84.1(8.2) | 87.6(6.8) | 87.7(6.6) | 87.4(6.8) | 0.177 |
| MCH | 29.7(2.5) | 29.8(2.3) | 29.8(2.4) | 28.4(2.9) | 28.3(3.0) | 26.2(6.8) | 29.5(2.7) | 29.5(2.6) | 29.5(2.7) | 0.096 |
| MCHC | 33.7(1.0) | 33.8(0.8) | 33.8(0.9) | 33.4(0.9) | 33.3(0.9) | 33.3(1.0) | 33.7(0.8) | 33.6(0.9) | 33.7(0.9) | 0.694 |
| RDW | 14(1.1) | 14(1.1) | 14.1(1.3) | 14.6(1.8) | 14.6(1.7) | 14.5(1.8) | 13.8(0.9) | 13.8(0.8) | 14(0.8) | 0.447 |
| Platelet Count | 219(56.1) | 217(57.3) | 234(67.8) | 237(43.5) | 240(44.7) | 230(40.2) | 237(44.1) | 227(39.7) | 231(42.7) | 0.056 |
| MPV | 8.6(0.7) | 8.6(0.7) | 8.5(0.6) | 8.8(1.0) | 8.7(0.8) | 8.7(0.9) | 8.8(1.1) | 8.6(1.1) | 8.6(1.1) | 0.887 |
| Neutrophils % | 58.8(7.5) | 57.1(7.8) | 54.5(5.8) | 59.6(8.3) | 60.7(8.0) | 62.3(11.1) | 56.7(8.0) | 53.1(8.5) | 54.5(9.1) | 0.015* |
| Lymphs % | 30.1(8.1) | 30.5(7.9) | 32.7(5.7) | 28.9(8.7) | 27.6(8.6) | 27.7(8.6) | 30.5(8.3) | 34.5(8.4) | 32.8(9.3) | 0.052 |
| Basos % | 0.7(0.3) | 0.7(0.3) | 0.7(0.4) | 0.8(0.3) | 0.7(0.2) | 0.7(0.3) | 0.7(0.3) | 0.7(0.3) | 0.7(0.2) | 0.323 |
| Monocytes % | 8.1(1.7) | 8.9(1.9) | 9.1(2.0) | 7.9(2.3) | 8.4(2.4) | 8.5(2.3) | 8.9(2.5) | 8.7(1.7) | 9(1.9) | 0.238 |
| Eos % | 2.4(1.2) | 2.9(1.5) | 3.0(1.9) | 2.7(1.4) | 2.6(1.0) | 2.6(1.3) | 3.0(1.7) | 2.8(1.6) | 3.1(1.4) | 0.145 |
| Neutrophils Absolute | 3.2(1.0) | 3(1.0) | 2.9(0.9) | 3.6(0.9) | 3.6(1.0) | 3.5(0.8) | 3.4(1.3) | 2.9(0.9) | 3.2(1.5) | 0.228 |
| Lymphocytes Absolute | 1.6(0.6) | 1.6(0.7) | 1.7(0.5) | 1.7(0.6) | 1.6(0.6) | 1.6(0.7) | 1.8(0.6) | 1.8(0.6) | 1.8(0.7) | 0.386 |
| Monocytes Absolute | 0.4(0.1) | 0.5(0.1) | 0.5(0.1) | 0.5(0.1) | 0.5(0.2) | 0.5(0.1) | 0.9(1.7) | 0.5(0.1) | 0.5(0.1) | 0.908 |
| Eosinophils Absolute | 0.1(0.1) | 0.2(0.1) | 0.2(0.1) | 0.2(0.1) | 0.2(0.1) | 0.2(0.1) | 0.2(0.1) | 0.2(0.1) | 0.2(0.1) | 0.09 |
| Basophils Absolute | 0.03(0.02) | 0.04(0.03) | 0.04(0.02) | 0.05(0.03) | 0.04(0.02) | 0.04(0.02) | 0.04(0.02) | 0.04(0.02) | 0.04(0.02) | 0.087 |

*Note: Mean(SD); NR+Ex: NR combined with exercise; PL+Ex: placebo combined with exercise; p-value was derived from comparisons of the mean change (6-week value minus baseline) between groups, statistical significance (p<0.05) marked with**
